# Nonuniformity of Whole-Cerebral Neural Resource Allocation, a Neuromarker of the Broad-Task Attention

**DOI:** 10.1523/ENEURO.0358-21.2022

**Published:** 2022-03-14

**Authors:** Jinyong Chung, Peter Lee, Young-Beom Lee, Kwangsun Yoo, Yong Jeong

**Affiliations:** 1 Department of Psychology, Yonsei University, Seoul 03722, Republic of Korea; 2 Department of Bio and Brain Engineering, Korea Advanced Institute of Science and Technology for Health Science and Technology, Daejeon 34141, Republic of Korea; 3 Center for Cognition and Sociality, Institute for Basic Science, Daejeon 34126, Republic of Korea; 4 Department of Psychology, Yale University, New Haven, Connecticut 06520-8205

**Keywords:** attention, functional magnetic resonance imaging, neural resource allocation

## Abstract

The neural basis of attention is thought to involve the allocation of limited neural resources. However, the quantitative validation of this hypothesis remains challenging. Here, we provide quantitative evidence that the nonuniform allocation of neural resources across the whole cerebral gray matter reflects the broad-task process of sustained attention. We propose a neural measure for the nonuniformity of whole-cerebral allocation using functional magnetic resonance imaging. We found that this measure was significantly correlated with conventional indicators of attention level, such as task difficulty and pupil dilation. We further found that the broad-task neural correlates of the measure belong to frontoparietal and dorsal attention networks. Finally, we found that patients with attention-deficit/hyperactivity disorder showed abnormal decreases in the level of the proposed measure, reflecting the executive dysfunction. This study proposes a neuromarker suggesting that the nonuniform allocation of neural resources may be the broad-task neural basis of sustained attention.

## Significance Statement

Quantitative evidence for the neural basis of attention, which is thought to involve neural resource allocation, is still lacking. Here, we propose a neural measurement quantifying the nonuniformity of whole-cerebral resource allocation. The level of the measure had positive linear relationships with indicators of attention level, such as task difficulty and pupil dilation. The cross-task and cross-dataset validations suggest that the measure could be used as a neuromarker of broad-task sustained attention. Its broad-task neural correlates belong to frontoparietal and dorsal attention networks. We further explored levels of the measure in patients with attention-deficit/hyperactivity disorder and observed abnormal decreases reflecting their executive dysfunctions compared with healthy individuals. This result underlines the utility of the measure as a neuromarker.

## Introduction

The limited resources for mental processing in the human brain constrain cognitive behaviors (Luck and Vogel, 1997; Marois and Ivanoff, 2005). The brain overcomes this by allocating limited resources efficiently and flexibly, depending on task demands (Raichle, 2006; Peters, 2011). Sustained attention, defined as the cognitive-behavioral process of maintaining concentration on specific information while ignoring other perceivable information, has been considered evidence of the allocation process of limited resources in the brain (Anderson, 2004; Nobre and Kastner, 2014). Studies have shown both task-general (Giesbrecht et al., 2003; Macaluso et al., 2003; Greenberg et al., 2010) and task-specific (Kanwisher and Wojciulik, 2000; Fries et al., 2001; Gazzaley et al., 2005; Falkner et al., 2010; Störmer and Alvarez, 2014; Moore and Zirnsak, 2017) characteristics. Common neural substrates for attention during various tasks have indicated that there may be a central neural process in charge of the broad-task process of sustained attention. However, the neural basis of this process remains unclear.

Previously published theories have suggested that the neural basis of attention is the allocation process of limited resources and have conceptually modeled this process (Moray, 1967; Kahneman, 1973; Wickens, 1980; Luck et al., 1996; Wickens, 2002; Watanabe and Funahashi, 2014). A capacity model of attention (Kahneman, 1973) provides an organized theory to address various aspects of attention. The model includes an allocation policy that achieves a nonuniform allocation of the available capacity in a task-relevant manner. Previous studies have reported evidence of the nonuniform allocation phenomenon caused by attention (Gazzaley et al., 2005; Falkner et al., 2010; Störmer and Alvarez, 2014). The representative neural features of attention are attentional enhancement and surround suppression. Neural activity encoding task-relevant or attended stimuli is enhanced, whereas neural activity associated with task-irrelevant or unattended stimuli is suppressed (Gazzaley et al., 2005; Falkner et al., 2010; Störmer and Alvarez, 2014). These findings indicate that limited neural resources are allocated in a task-relevant manner, resulting in nonuniform allocation. Furthermore, in large-scale nonuniform allocation, it also has been observed that neural activities of task-irrelevant networks are inhibited while task-relevant network activities are enhanced (Vincent et al., 2008; Hermans et al., 2014). The same consequence of nonuniform allocations in local and large-scale levels suggests there may be a global process controlling resource allocation across the whole brain. Although this qualitative evidence supports the hypothesis of the neural basis of attention, quantitative evidence is still lacking. The absence of a quantitative measure for nonuniform resource allocation is a significant obstacle in the quantitative validation of this hypothesis.

Here, we aim to provide quantitative evidence that the nonuniform allocation of limited neural resources reflects the broad-task process of sustained attention. We propose a neuroimaging measure that quantifies the nonuniformity of whole-cerebral neural resource allocation (nu-NRA). Using this measure, we tested the following hypotheses: (1) nonuniform allocation of neural resources across the whole cerebral gray matter reflects the broad-task sustained attention; (2) broad-task neural substrates of nonuniform resource allocation may exist; and (3) alterations in the levels of the measure are observed in patients with attention-deficit/hyperactivity disorder (ADHD) compared with healthy individuals.

We first validated the measure using task difficulty reflecting attentional load, an experimental indicator of attention level (Sunaert et al., 2000; Culham et al., 2001; Chen et al., 2008). If the attentional load was controlled without changing types of stimuli and tasks, task difficulty well follows attentional load. We further explored the quantitative relationships of this new measure with pupil dilation, which is a physiological indicator (Verney et al., 2004; Wierda et al., 2012; Alnæs et al., 2014). We then performed a cross-task and cross-dataset validation and investigated the neural correlates of the measure by brain mapping. Finally, we explored alterations in the nu-NRA levels in patients with ADHD compared with healthy individuals.

## Materials and Methods

### Datasets

#### Dataset 1

Functional and structural magnetic resonance (MR) images were acquired using a 3 T MR scanner (Magnetom Verio, Siemens) equipped with a 32-channel head coil at the Korea Advanced Institute of Science and Technology fMRI Center in Daejeon, Republic of Korea. Blood oxygenation-level-dependent (BOLD) measurements for a task and resting-state functional MR imaging (fMRI) were performed using a gradient echo (GE) echoplanar imaging (EPI) sequence (repetition time (TR), 2000 ms; echo time (TE), 30 ms; slice thickness, 3 mm; field of view (FOV), 192 × 192 × 108 mm; flip angle (FA), 90°; voxel size, 3 × 3 × 3 mm; 36 axial slices with interleaved-ascending order]. T1-weighted structural MR images were collected using a three-dimensional magnetization-prepared rapid acquisition GE (3D-MPRAGE) sequence (TR, 1800 ms; TE, 2.52 ms; FOV, 256 × 256 × 176 mm; FA, 9°; voxel size, 1 × 1 × 1 mm; 176 sagittal slices).

Twenty-four participants (four women; age range, 19–34 years; mean age, 25 years; all right handed) were recruited. None of the participants had any history of neurologic or psychiatric illness, and all had 20/20 vision. One participant was excluded from analyses because the upper part of the brain was outside the field of view of the fMRI. Task performance and physiological data were missing for seven participants and were, thus, excluded from the corresponding analyses. The present study was approved by the Institutional Review Board of the Korea Advanced Institute of Science and Technology. Participants were provided with monetary compensation commensurate with task performance.

The visuospatial *n*-back working memory task was designed to induce various attentional load levels without changing the stimuli. We implemented this task using Psychtoolbox-3 (www.psychtoolbox.org). Stimuli were projected onto the participants’ eyes through a head-mounted display (NordicNeuroLab VisualSystem HD). Red, green, or blue circles were used as stimuli, with the color representing the target feature. Each color was first set to the maximum RGB level [e.g., red, (255, 0, 0)], and the luminance was reduced by half to decrease visual fatigue during the tasks [red, (165, 0, 0); green, (0, 130, 0); blue, (0, 0, 212)]. The circles were presented at 4.5° eccentricity (radius, 1.5°) to the left or right of the central fixation point. Throughout all tasks, participants were instructed to focus their eyes consistently on the central fixation cross. During the *n*-back task, participants were required to memorize the color of the previous *n*th stimuli and match it with the present target on the attended side. Participants performed 1-back, 2-back, and 3-back tasks while attending to left or right stimuli. After a 30 s fixation block, task cues were presented for 5 s, followed by a 1 s delay. The cues consisted of a number representing the *n*-back type and an eye icon. The left *n*-back task began after 3 s of the “left” spatial cue presentation and a 1 s delay. After the left *n*-back task, the right *n*-back task began following the presentation of a “right” spatial cue. Each spatial *n*-back task consisted of 40 trials, and each trial included 2 s of stimulus presentation followed by a 0.5 s delay. Participants were able to respond regarding whether the present stimuli matched the previous *n*th target during this 2.5 s period. Fifteen or 16 “yes” trials were pseudorandomly assigned to each spatial *n*-back task. In addition, we included a passive-viewing task beginning with a “P” task cue as a control for the *n*-back task, using the same visual stimuli and task design but requiring no working memory load. Participants were not instructed to memorize anything or pay spatial attention following spatial cues during the passive-viewing task. We also acquired 3 min of resting-state fMRI (eyes open) and structural T1 MRI data before the task fMRI.

#### Dataset 2

Dataset 2 was a subset of the Human Connectome Project (HCP) database. Functional imaging data obtained during the resting state (eyes open) and five cognitive tasks (working memory, emotion, gambling, relational, and social) were selected for cross-task and cross-dataset validation. All tasks have been described in detail previously (Barch et al., 2013). Whole-brain EPIs were acquired using a 3 T MR scanner (Skyra, Siemens) equipped with a 32-channel head coil (TR, 720 ms; TE, 33.1 ms; FOV, 208 × 180 × 72 mm; FA, 52°; 2.0 mm isotropic voxels). Two runs for each resting-state and task fMRI were acquired, with right-to-left and left-to-right phase encoding. All analyses were performed separately for each phase encoding and then averaged. Structural T1 MRI data were also acquired using a 3D-MPRAGE sequence (TR, 2400 ms; TE, 2.14 ms; FOV, 224 × 320 mm; FA, 8°; 0.7 mm isotropic voxels). Information regarding the data acquisition and processing pipelines has been provided in detail previously (Glasser et al., 2013; Van Essen et al., 2013).

We selected 98 participants (68 women; age range, 22–35 years), referring to the list of HCP participants provided by Tavor et al. (2016). Minimally preprocessed structural MRI and fMRI data were used. The Human Connectome Project dataset was downloaded from https://db.humanconnectome.org.

#### Dataset 3

Dataset 3 was a subset of the Consortium for Neuropsychiatric Phenomics database (Poldrack et al., 2016; Bilder et al., 2020). Functional and structural MR images were acquired using a 3 T MR scanner (Trio, Siemens). Functional MRI data were collected using an EPI sequence (TR, 2000 ms; TE, 30 ms; slice thickness, 3 mm; FOV, 64 × 64 × 192 mm; FA, 90°; 34 axial slices with 4 mm thickness). T1-weighted structural MR images were collected using a 3D-MPRAGE sequence (TR, 1900 ms; TE, 2.52 ms; slice thickness, 1 mm; FOV, 256 × 256 × 250 mm; 176 sagittal slices).

We selected 106 healthy individuals (50 women; age range, 21–50 years; mean age, 32 years; all right handed) and 34 patients with ADHD (19 women; age range, 21–50 years; mean age, 35 years; all right handed) with no aliasing artifacts on the anatomic images and performing all tasks. We further excluded two subjects from the ADHD group because of uncertain ADHD clinical diagnostic scale information. The resting-state (eyes open) and spatial capacity working memory task fMRI data were analyzed.

During the task, participants were required to memorize an array of one, three, five, or seven circles (Loads 1–7) pseudorandomly positioned around a central fixation cross and were asked to respond whether the target circle was in the same position as one of the arrays. Half of the trials were “yes” trials, whereas the remaining half were “no” trials. The task design has been described in detail previously (Poldrack et al., 2016).

### Preprocessing steps

All preprocessing steps were performed using SPM12 (Wellcome Trust Center for Neuroimaging) and in-house codes in MATLAB R2018a (MathWorks). Functional scans acquired during the first 6 s were discarded to allow for equilibration effects (three scans for Datasets 1 and 3 with 2 s TR; 9 scans for Dataset 2 with 0.72 s TR). Slice-timing correction and spatial realignment to the first scan were achieved via rigid body transformation, following which images were spatially coregistered to T1 MRI scans. Images were then spatially normalized to the Montreal Neurologic Institute space (International Consortium for Brain Mapping) and spatially smoothed using a 4 mm full-width at half-maximum Gaussian kernel. Raw fMRI scans from Dataset 2, which had already been normalized, entered the smoothing step immediately. Brain tissues were then segmented using a normalized T1 MRI scan. Voxels with the highest corresponding probability values were assigned to each tissue mask (gray matter, white matter, CSF, soft tissue, and bone). We excluded cerebellar regions from the masks using an automated anatomic labeling template (Tzourio-Mazoyer et al., 2002). Subsequently, the fMRI scans underwent noise reduction steps. First, motion correction was performed to regress out head motion effects using the motion parameters acquired from the realignment step (6 parameters for Datasets 1 and 3; 12 parameters for Dataset 2). Then, they were passed through a high-pass filter at 0.008 Hz. Finally, we regressed out the white matter, CSF, soft tissue, and bone signals, while linear and quadratic temporal trends were also considered nuisance parameters.

### Assumptions and computation of the nu-NRA

We first assumed that fMRI is a suitable method for measuring the quantity of neural resources used in the brain. This method measures the BOLD signal reflecting blood oxygenation coupled to underlying neuronal demand by neurovascular coupling (Raichle, 1998; Hillman, 2014). We then assumed that brain regions need some neural resources to maintain the default mode of the brain; this would differ across regions. We considered the resting state as the default mode of the brain (Raichle et al., 2001; Greicius et al., 2003; Fransson, 2005; Buckner et al., 2008), reflecting default neural resource utilization. We averaged BOLD signals across resting-state scans (rsBOLD) to extract the average default utilization for each participant. This was finally *z*-normalized across voxels to standardize the BOLD signal amplitudes, which are not comparable across sessions and participants. The default utilization $\left(U_{v,D}\right)$ was calculated as follows:

$$u_{v,D}=\frac{1}{S}\overset{S}{\underset{s=1}{\sum }}{\text{rsBOLD}}_{v,s}$$

$$U_{v,D}=\frac{u_{v,D}-\frac{1}{V}\sum_{v=1}^{V}u_{v,D}}{\sqrt{\frac{\sum_{v=1}^{V}{\left(u_{v,D}-\frac{1}{V}\sum_{v=1}^{V}u_{v,D}\right)}^{2}}{V-1}}},$$

where v and s index the voxels and scans, and V and S represent the number of voxels and scans, respectively.

**Figure 1.**
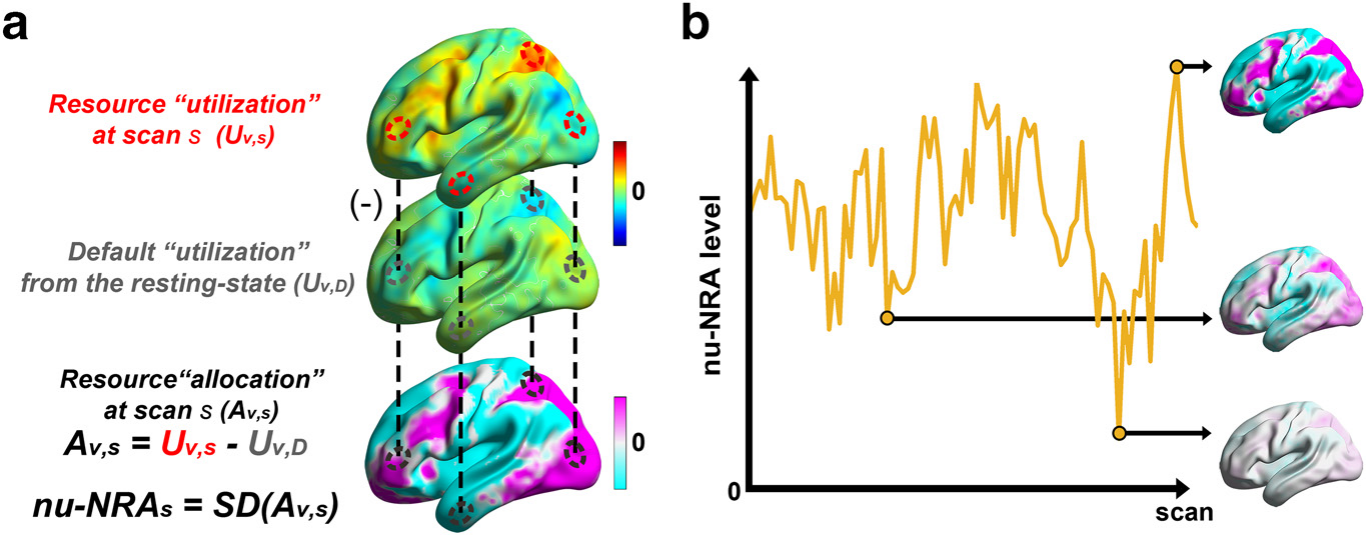
Definition of resource allocation and the nu-NRA. ***a***, The amount of neural resource allocation $\left(A_{v,s}\right)$ is defined as the change in neural resource utilization $\left(U_{v,s}\right)$ at each task scan from the default utilization $\left(U_{v,D}\right)$. ***b***, We defined the nu-NRA by calculating the spatial SD across the whole-cerebral resource allocation at each task scan to quantify the nonuniformity of resource allocation. A higher level of the measure indicates a brain state with more nonuniform resource allocation across the whole cerebral gray matter.

Based on the default resources assumption, we defined the amount of allocated neural resources caused by task demands as the change from the default utilization (Fig. 1*a*). At each task scan, we z-normalized BOLD signals across voxels to extract resource utilization $\left(U_{v,s}\right)$. This normalized signal subtracts out the default utilization $\left(U_{v,D}\right)$, and the resulting value is assigned to the amount of allocation $\left(A_{v,s}\right)$ at the task scan s. The amount of resource allocation $\left(A_{v,s}\right)$ is calculated as follows:

$$U_{v,s}=\frac{{\text{BOLD}}_{v,s}-\frac{1}{V}\sum_{v=1}^{V}{\text{BOLD}}_{v,s}}{\sqrt{\frac{\sum_{v=1}^{V}{\left({\text{BOLD}}_{v,s}-\frac{1}{V}\sum_{v=1}^{V}{\text{BOLD}}_{v,s}\right)}^{2}}{V-1}}}$$

$$A_{v,s}=U_{v,s}-U_{v,D}.$$

Finally, we defined the nu-NRA to quantify the nonuniformity of neural resource allocation across the whole cerebral gray matter (Fig. 1*b*). We calculated the nonuniformity based on the spatial SD of the resource allocation $\left(A_{v,s}\right)$ as follows:

$$\text{nu}-{\text{NRA}}_{s}=\sqrt{\frac{\sum_{v=1}^{V}(A_{v,s}-\frac{1}{V}\sum_{v=1}^{V}A_{v,s})}{V-1}}.$$

We computed a raw measure within the segmented cerebral gray matter mask using the high pass-filtered fMRI scans. The raw measure then underwent a nuisance regression step similar to the fMRI preprocessing to be a final nu-NRA. We regressed out nu-NRA signals within the other tissues.

### Assessing pupil dilation

Dataset 1 included pupil information acquired using an eye-tracking camera (60 Hz) equipped with the VisualSystem HD (NordicNeuroLab). Both eyes were not always well detected because of the pupillary distance variance across the participants. Previous studies have shown that left and right pupil sizes are positively correlated during tasks (Blumenfeld, 2002; Foroughi et al., 2017). Thus, we manually selected the eye with the better detection rate in evaluating the number of misdetection events and used it for the analysis. Pupil height and width time series were low-pass filtered (third-order Butterworth; cutoff, 4 Hz). The pupil diameter was determined by averaging the height and width. Pupil dilation for each trial was calculated based on the percent changes in the average pupil diameter during the trial from the average in the preceding spatial cue block (4 s). We excluded outliers using the interquartile method, thresholding at 1.5 times the interquartile range.

### Brain mapping of the nu-NRA

We created an individual map by calculating voxelwise Pearson correlations between the measure and BOLD signal time series for each participant and task. Correlations were transformed into *z* scores via Fisher’s *z*-transformation. We then applied a voxelwise one-sample *t* test across individual maps, and the resulting *t* scores constructed a group map for each task. The statistical significance of the spatial similarities among the group maps was assessed using a randomization procedure. We permuted nu-NRA time courses and performed brain mapping within individuals. One hundred permuted group maps were extracted for each task. All permuted spatial similarities among the tasks were collapsed into a randomization distribution to correct multiple comparisons. Finally, we explored broad-task neural substrates by finding overlapping voxels with significant [false discovery rate (FDR)-corrected, *p* < 0.05; Benjamini and Hochberg, 1995; Groppe et al., 2011] positive correlations across all tasks. We rendered volumetric results of brain mapping onto the brain surface using BrainNet Viewer (Xia et al., 2013).

### Data availability

Dataset 1 and a code for the nu-NRA computation are freely available online at https://data.mendeley.com/datasets/7ydmfmk8kt/2. The code is available as Extended Data 1.

10.1523/ENEURO.0358-21.2022.ed1 Extended Data 1 Supplementary Code_Data_Accessibility. Download Extended Data 1, DOCX file.

## Results

### Task performance and level of the nu-NRA during the visuospatial *n*-back working memory task

**Figure 2.**
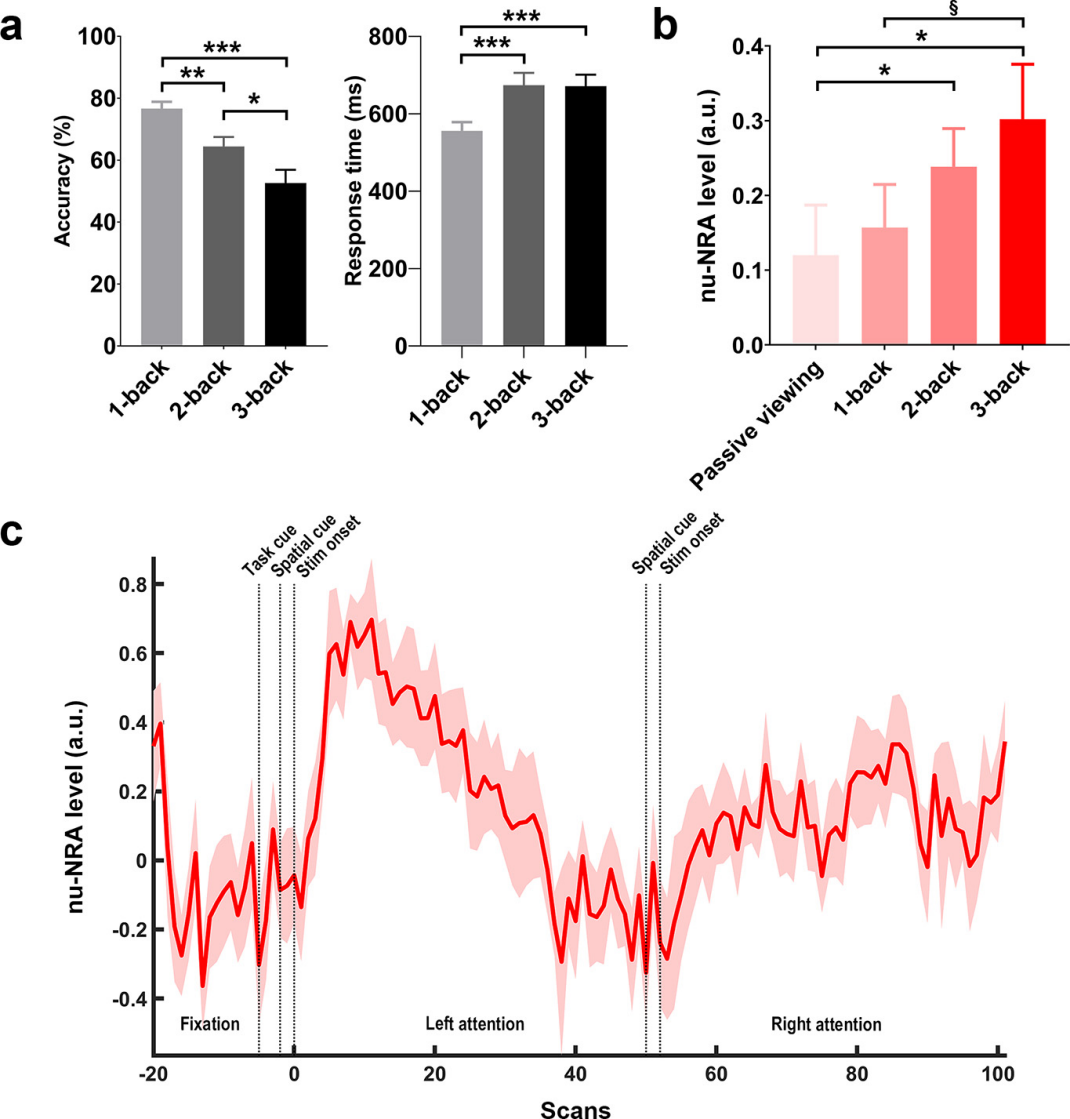
Task performance and the level of the nu-NRA during the visuospatial *n*-back working memory task in Dataset 1. ***a***, Left, Task accuracy across conditions. Task accuracies gradually decrease from the 1-back to the 3-back task. Right, Response times across conditions. Response times are longer in the 2-back and 3-back tasks than in the 1-back task. ***b***, The nu-NRA level across conditions. Levels gradually increase from the passive-viewing task to the 3-back task (Extended Data Fig. 2-1, results on network activations). ***c***, The temporal dynamics of the nu-NRA during the *n*-back task. The graph is a group-averaged time series of the nu-NRA during all *n*-back tasks collapsed into one graph. The shaded region indicates cross-individual variance (SE). §*p* < 0.08, *p < 0.05, **p < 0.01, ***p < 0.001. Error bars indicate SEM values.

Task performances were compared across conditions (Fig. 2*a*). Task accuracies were different (repeated-measures ANOVA: *F*_(2,14)_ = 14.529, *p* < 0.0001, partial η^2^ = 0.66), and a gradual decrease in accuracy was observed from 1-back to 3-back tasks [within-participant contrast (linear trend): *F*_(1,15)_ = 27.261, *p* < 0.001, partial η^2^ = 0.65]. *Post hoc* pairwise *t* tests confirmed the decrease (1-back vs 2-back: *t*_(15)_ = 3.153, *p* < 0.01; 2-back vs 3-back: *t*_(15)_ = 2.440, *p* < 0.05). Response times were also different across these conditions (repeated-measures ANOVA: *F*_(2,14)_ = 9.006, *p* < 0.01, partial η^2^ = 0.69), and the response times in the 2-back and 3-back tasks were longer than those in the 1-back task (1-back vs 2-back: *t*_(15)_ = −4.397, *p* < 0.001, Cohen’s *d* = −0.79; 1-back vs 3-back: *t*_(15)_ = −3.987, *p* < 0.001, Cohen’s *d* = −0.63). This result indicates that the task well introduces different loads for the participants across the conditions.

10.1523/ENEURO.0358-21.2022.f2-1 Figure 2-1 Network activations during the visuospatial *n*-back working memory task in Dataset 1. *p < 0.05, **p < 0.01, ***p < 0.001. Download Figure 2-1, TIF file.

We then evaluated the levels of the nu-NRA across the conditions (Fig. 2*b*). We *z*-normalized levels within the conditions using the mean and SD of that from all fixation and cue blocks for each participant. We assigned the averaged levels to the conditions with a delay of three scans (6 s) by considering the hemodynamic response lag (Liao et al., 2002). As expected, the levels of the nu-NRA gradually increased from the passive-viewing condition to the 3-back task (repeated-measures ANOVA: *F*_(3,13)_ = 3.151, *p* < 0.05, partial η^2^ = 0.43; within-participant contrast (linear trend): *F*_(1,15)_ = 8.998, *p* < 0.01, partial η^2^ = 0.38]. *Post hoc* pairwise *t* tests confirmed the significant differences between increases (passive viewing vs 2-back: *t*_(15)_ = −2.346, *p* < 0.05, Cohen’s *d* = −0.55; passive viewing vs 3-back: *t*_(15)_ = −2.626, *p* < 0.05, Cohen’s *d* = −0.61; 1-back vs 3-back: *t*_(15)_ = −1.947, *p* = 0.071, Cohen’s *d* = −0.44). This indicates that the level of the measure is greater when participants experience greater task difficulty.

We further explored the time courses of the nu-NRA during the tasks. The measure was collapsed across all *n*-back tasks. We observed task-relevant changes in nu-NRA levels, which were lowest during fixation and increased after three scans (i.e., 6 s) following stimulus onset. Gradual decreases were observed in the first block (left attention), but the overall levels during the tasks were higher than those during fixation and cue periods. These findings are similar to the typical dynamics of neural activation during sustained attention (Visscher et al., 2003; Dosenbach et al., 2006; Petersen and Dubis, 2012), which is represented by the activity associated with task initiation and maintenance (Fig. 2*c*). This suggests that the nu-NRA may reflect the attention level during a visuospatial *n*-back working memory task.

### Relationship between nu-NRA level and pupil dilation

**Figure 3.**
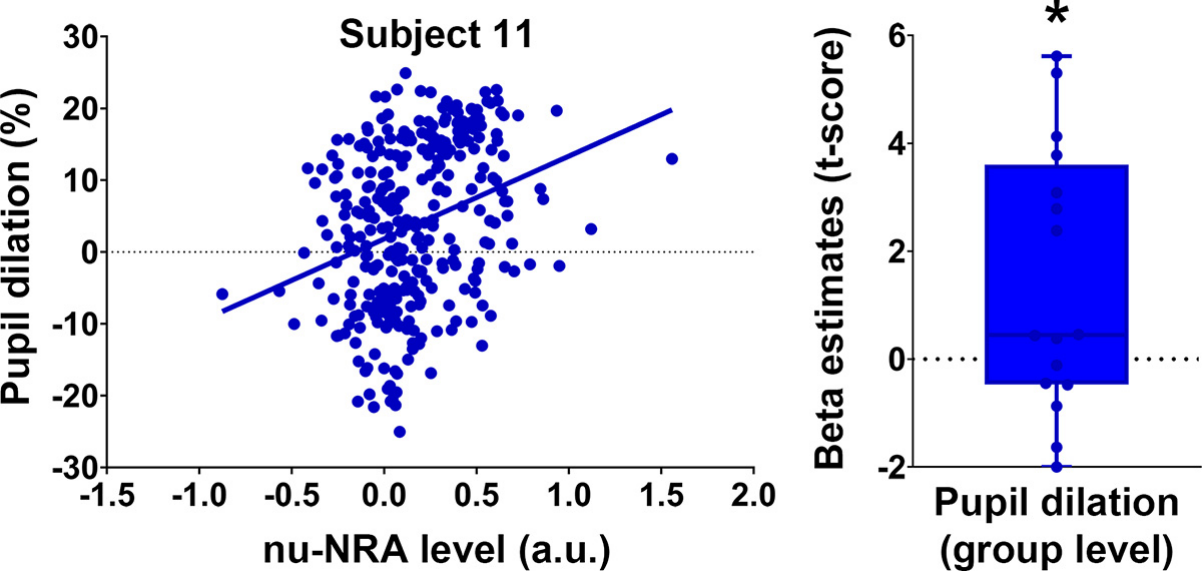
Relationship between the nu-NRA and pupil dilation. Individual- and group-level analyses between nu-NRA and pupil dilation. The representative subject (subject 11) shows the highest positive correlation among all study participants. The group-level result confirmed the relationship (Extended Data Fig. 3-1, results on network activations). β-Estimates are linear terms of regression coefficients. They were transformed into *t* scores for the analysis. *p < 0.05, **p < 0.01, ***p < 0.001. Error bars indicate SEM values.

We also investigated whether the nu-NRA exhibits quantitative relationships with the physiological indicator of attention level. The amount of pupil dilation is a well known physiological indicator of attention and cognitive control. The pupil dilates as a function of attentional load or mental efforts (Verney et al., 2004; Wierda et al., 2012; Alnæs et al., 2014). We first performed a within-participant linear regression between pupil dilation and the nu-NRA for all trials collapsed across task conditions. The nu-NRA level for each trial was calculated by interpolation and averaged from the scan series, considering the hemodynamic response lag of 6 s. We removed outliers using Cook’s distance of <*n*/4 (Cook, 1977; Hadi and Simonoff, 1993). We performed nonparametric tests for a group-level analysis on the *t* scores of β estimates (linear term). We shuffled pupil diameters across nu-NRA levels within participants. We repeated the shuffling 5000 times and calculated a permutation *p*-value for the group statistic. The regression of the most significant participants and a group-level result is shown in Figure 3. The nu-NRA was positively correlated with pupil dilation (permutation *p* < 0.05). This result further supports the notion that the nu-NRA may quantitatively reflect the attention level.

10.1523/ENEURO.0358-21.2022.f3-1 Figure 3-1 Relationship between network activations and pupil dilation. *p < 0.05, **p < 0.01, ***p < 0.001. Download Figure 3-1, TIF file.

### Cross-task and cross-dataset validation of the nu-NRA

**Figure 4.**
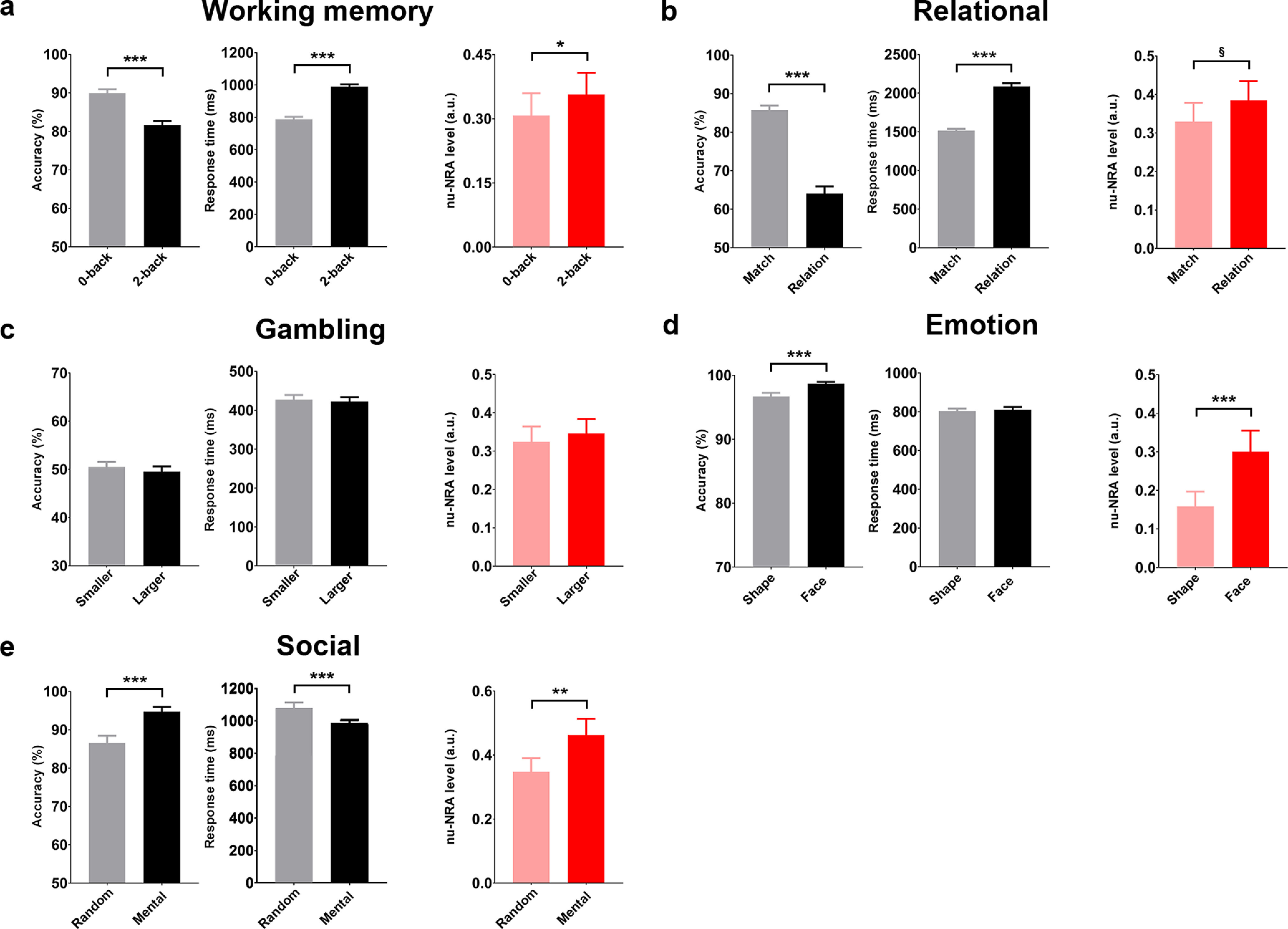
Levels of the nu-NRA across task conditions in Dataset 2 (Extended Data Fig. 4-1, results of network activations). ***a***, The 2-back working memory task shows a higher level of the measure with greater task difficulty and longer response time than the 0-back task. ***b***, The relational task shows a higher level of the measure with greater task difficulty and longer response time than the matching task. ***c***, There is no difference in the nu-NRA level between smaller and larger conditions with no difference in task difficulty and response time. ***d***, The task with face stimuli shows a higher level of the measure with lower task difficulty than the task with shape stimuli. ***e***, The mental condition with socially interacting stimuli shows a higher level of the measure with lower task difficulty and shorter response time than the condition with randomly moving stimuli. §*p* < 0.08, *p < 0.05, **p < 0.01, ***p < 0.001. Error bars indicate SEM values.

We first evaluated the nu-NRA levels during the working memory task in Dataset 2 (Fig. 4*a*). The level was higher (paired *t* test: *t*_(97)_ = 2.07, *p* < 0.05, Cohen’s *d* = 0.21) during the 2-back task with greater task difficulty (paired *t* test: *t*_(97)_ = −7.54, *p* < 10^−10^, Cohen’s *d* = −0.76) and longer response time (paired *t* test: *t*_(97)_ = 14.63, *p* < 10^−10^, Cohen’s *d* = 1.48) compared with the 0-back task. This corresponds with the result from Dataset 1, demonstrating that the level of the measure reflects task difficulty regardless of the dataset. We also explored the levels of the nu-NRA during the other tasks from Dataset 2. The level was higher, although the statistical significance was marginal (paired *t* test: *t*_(97)_ = 1.80, *p* = 0.075, Cohen’s *d* = 0.18), during the relation condition with greater task difficulty (paired *t* test: *t*_(97)_ = 12.81, *p* < 10^−10^, Cohen’s *d* = 1.29) and longer response time (paired *t* test: *t*_(97)_ = 18.2, *p* < 10^−10^, Cohen’s *d* = 1.84) compared with the matching condition, as we expected (Fig. 4*b*). During the gambling task, there was no level difference between conditions with different guesses (Fig. 4*c*). This also matches the expectation because the conditions have the same task and stimulus, except for the response. However, the levels of the nu-NRA did not always follow the task difficulty when the stimulus was different across conditions (Fig. 4*d*,*e*). The emotion (paired *t* test for accuracy: *t*_(97)_ = −3.52, *p* < 0.001, Cohen’s *d* = −0.38) and social tasks (paired *t* test for accuracy: *t*_(97)_ = −3.58, *p* < 0.001, Cohen’s *d* = −0.34) showed lower levels of the measure during the difficult conditions (paired *t* test for the emotion task: *t*_(97)_ = −3.76, *p* < 0.001, Cohen’s *d* = −0.36; paired *t* test for the social task: *t*_(97)_ = −3.38, *p* < 0.01, Cohen’s *d* = −0.36). Different conditions in these tasks used different types of stimuli instead of only controlling the attentional load. Thus, task difficulty reflecting the stimulus type rather than the attentional load may induce different results from the working memory, the relational, and the gambling task (see Discussion).

10.1523/ENEURO.0358-21.2022.f4-1 Figure 4-1 Network activations across task conditions in Dataset 2. *p < 0.05, **p < 0.01, ***p < 0.001. Download Figure 4-1, TIF file.

**Figure 5.**
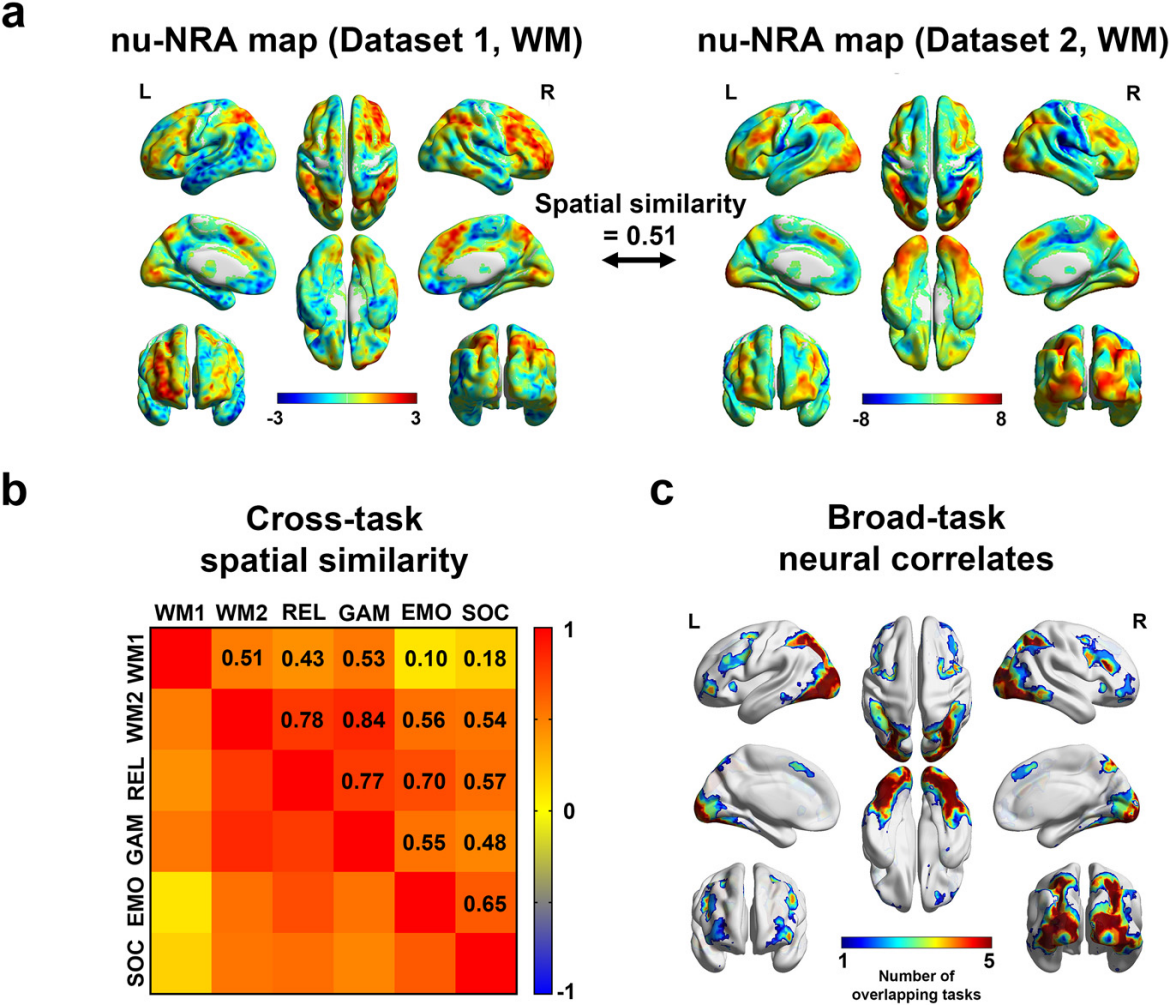
Brain mapping of the nu-NRA. ***a***, Brain mapping of the nu-NRA based on the working memory tasks in Datasets 1 and 2. Group nu-NRA maps are spatially similar across the datasets (Extended Data Fig. 5-3, comparison results). ***b***, Cross-task and cross-dataset comparisons between group nu-NRA maps. All pairs show significant similarities (Extended Data Fig. 5-1, the permutation test). Group nu-NRA maps for the other tasks in Dataset 2 are presented in Extended Data Figure 5-2. WM1, Working memory task in Dataset 1; WM2, working memory task in Dataset 2; EMO, emotion task in Dataset 2; GAM, gambling task in Dataset 2; REL, relational task in Dataset 2; SOC, social task in Dataset 2. ***c***, The broad-task neural correlates of nu-NRA are found by searching overlapping voxels with significant positive correlations across tasks (FDR-corrected, *p* < 0.05). L, left hemisphere; R, Right hemisphere.

We performed brain mapping of the nu-NRA to investigate the neural correlates across the cerebral gray matter. Group nu-NRA maps of working memory showed distinct regions with strong temporal correlations (Fig. 5*a*). They showed strong cross-dataset spatial similarity (*r* = 0.51). BOLD signals of the dorsal attention, frontoparietal, and visual networks (LaBar et al., 1999; Corbetta and Shulman, 2002; Fox et al., 2006; Ptak, 2012; Vossel et al., 2014) exhibit strong positive correlations with the measure, whereas the default-mode and sensory-motor networks (Lee et al., 2012) show strong negative correlations.

10.1523/ENEURO.0358-21.2022.f5-1 Figure 5-1 Randomization distribution for spatial similarities among the group nu-NRA maps. The mean value of permuted spatial similarities is near zero, and the maximum permuted similarity is near 0.11. The *p* value for the spatial similarity (*r* = 0.10) between the working memory task from Dataset 1 (WM1) and the emotion task from Dataset 2 (EMO) is <0.005. Download Figure 5-1, TIF file.

10.1523/ENEURO.0358-21.2022.f5-2 Figure 5-2 nu-NRA maps for four tasks in Dataset 2. ***a***, Relational task. ***b***, Gambling task. ***c***, Emotion task. ***d***, Social task. Download Figure 5-2, TIF file.

10.1523/ENEURO.0358-21.2022.f5-3 Figure 5-3 Comparison of the nu-NRA maps during the working memory tasks between Dataset 1 and 2. Positive (red) values indicate higher correlations (two-sample *t* tests, FDR-corrected, *p* < 0.05) in Dataset 1 than in Dataset 2. Dataset 1, presenting peripheral stimuli, shows higher correlations in medial parts of the primary visual cortex. By contrast, Dataset 2, presenting central object stimuli, shows higher correlations (blue) in the lateral parts of the primary visual cortex and object-responsive lateral occipital regions. Download Figure 5-3, TIF file.

We also performed cross-task and cross-dataset validation for brain mapping (Fig. 5*b*). All cross-task and cross-dataset spatial similarities between the group maps were positively significant (permutation *p*-value < 0.005 for the lowest value; Extended Data Fig. 5-1). However, there is a possibility that the significant similarities were solely achieved by visual regions that broadly and consistently showed positive similarities across the tasks (Extended Data Fig. 5-2). Thus, we explored broad-task neural correlates of the nu-NRA by searching overlapping regions, which showed significant positive correlations across the tasks (FDR-corrected, *p* < 0.05; Fig. 5*c*). We found bilateral parietal and right middle frontal regions, which belong to dorsal attention and frontoparietal networks, as well as visual network (LaBar et al., 1999; Corbetta and Shulman, 2002; Fox et al., 2006; Yeo et al., 2011; Ptak, 2012; Vossel et al., 2014). These results suggest that the bilateral parietal and right middle frontal regions may be the central neural substrates for the broad-task process of sustained attention controlling the nonuniformity of resource allocation.

However, the brain mapping results also suggested the possibility that the nu-NRA level could represent certain large-scale network activations rather than the nonuniformity of whole-brain resource allocation. To validate the possibility, we calculated network activations and tested them with the same analyses using the seven large-scale networks from the study by Yeo et al. (2011). The parcellation includes visual, somatomotor, dorsal attention, ventral attention, limbic, frontoparietal, and default mode networks (DMNs). As shown in Extended Data Figure 2-1, only the network activation of DMNs showed a linear trend (negative) along the task difficulty [repeated-measures ANOVA; attentional load: *F*_(3,13)_ = 5.31, *p* < 0.05, partial η^2^ = 0.55; within-participant contrast (linear trend): *F*_(1,15)_ = 9.506, *p* < 0.01, partial η^2^ = 0.39] in Dataset 1. Dorsal attention and frontoparietal network activation showed elevated levels during higher attentional loads but without statistical significance. The amount of pupil dilation was also not correlated with task-positive network activations (Extended Data Fig. 3-1). However, it was negatively correlated with the visual (permutation, *p* < 0.05) and somatomotor (permutation, *p* < 0.05) networks. It might be that large-scale sensory networks also include surrounding inhibition in task-unrelated sensory regions and focal excitation in task-relevant sensory regions. Also, as shown in Extended Data Figure 4-1, network activations partly followed the task difficulty with distinct patterns across the tasks in Dataset 2. Dorsal attention network activations followed the task difficulty only in the relational task (paired *t* test: *t*_(97)_ = 2.73, *p* < 0.01, Cohen’s *d* = 0.28) and the emotion task (paired *t* test: *t*_(97)_ = 5.08, *p* < 0.001, Cohen’s *d* = 0.51). Frontoparietal network activation followed the task difficulty during the working memory task (paired *t* test: *t*_(97)_ = 12.70, *p* < 0.001, Cohen’s *d* = 1.28) and relational task (paired *t* test: *t*_(97)_ = 9.25, *p* < 0.001, Cohen’s *d* = 0.93), but not during other tasks. There was no network in which activation followed the task difficulty in all tasks or followed the results from the level of nu-NRA. To sum up the results, the nu-NRA signal did not simply represent specific network activations but conveyed comprehensive information of all network activations.

### Levels of the nu-NRA in patients with ADHD

**Figure 6.**
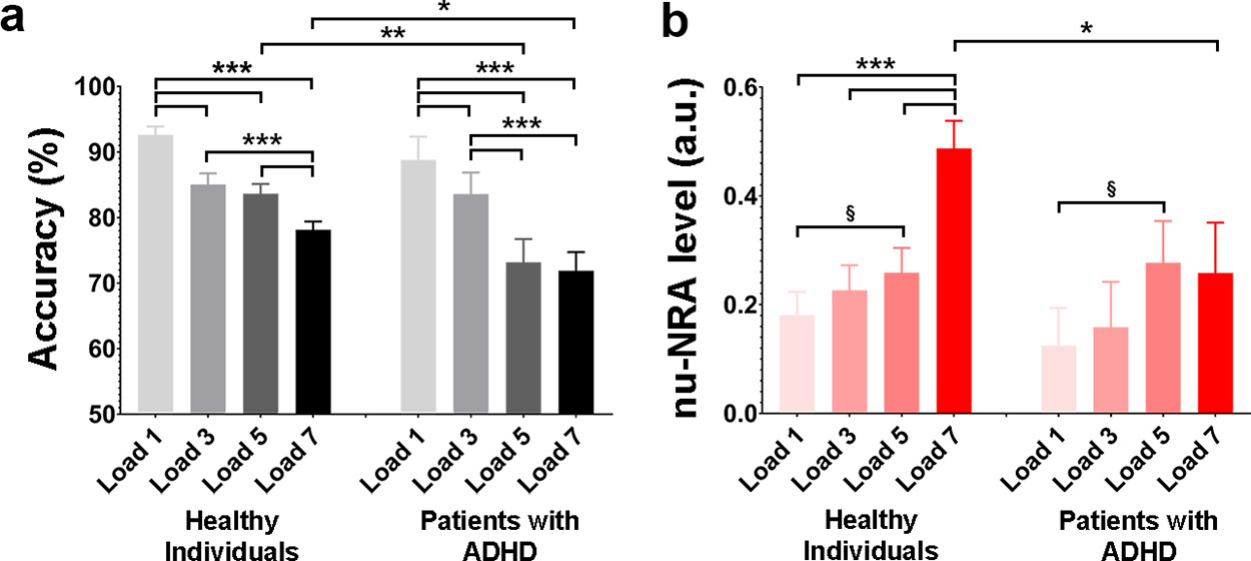
Task performance and level of the nu-NRA during spatial capacity working memory task in healthy individuals and patients with ADHD. ***a***, Task accuracy across the conditions. Task accuracies gradually decrease from Load 1 to Load 7 in both groups. The patients with ADHD show lower accuracy at Load 5 and Load 7 than the healthy individuals. ***b***, The nu-NRA level across conditions. In healthy individuals, levels gradually increase from Load 1 to Load 7 with rapid increases from Load 5 to Load 7. However, patients with ADHD do not show any level increase at Load 7. §*p* < 0.08, *p < 0.05, **p < 0.01, ***p < 0.001. Error bars indicate SEM values.

ADHD is a psychiatric disorder characterized by persistent inattention and/or hyperactivity-impulsivity that interferes with functioning in daily life (American Psychiatric Association, 2013). Many studies have demonstrated that patients with ADHD show executive dysfunctions because of alterations in the resource allocation process (Gualtieri and Johnson, 2006; Kratz et al., 2011; Dörrenbächer and Kray, 2019). We thus expected that patients with ADHD would experience difficulties allocating sufficient neural resources during a higher resource-demanding task. This may lead to a decrease in the level of the nu-NRA in this task. We first compared the task accuracy between healthy individuals and patients with ADHD using the spatial capacity working memory task (Dataset 3). Both groups showed a gradual decrease in task accuracy from Load 1 to Load 7 [repeated-measures ANOVA; healthy individuals: *F*_(3,103)_ = 51.76, *p* < 10^−10^, partial η^2^ = 0.60; within-participant contrast (linear trend): *F*_(1,105)_ = 118.11, *p* < 10^−10^, partial η^2^ = 0.53; repeated-measures ANOVA; patients with ADHD: *F*_(3,29)_ = 18.96, *p* < 10^−6^, partial η^2^ = 0.66; within-participant contrast (linear trend): *F*_(1,31)_ = 58.58, *p* < 10^−7^, partial η^2^ = 0.65]. The patients with ADHD exhibited no task accuracy difference at Load 1 and Load 3, but lower accuracy at Load 5 and Load 7 (two-sample *t* test: *t*_(136)_ = −3.16, *p* < 0.01, Cohen’s *d* = −0.64; *t*_(136)_ = −2.26, *p* < 0.05, Cohen’s *d* = −0.46, respectively) compared with the healthy individuals (Fig. 6*a*). We then explored the levels of the nu-NRA during the delayed period between the target offset and probe onset (Fig. 6*b*). The nu-NRA level tended to increase with increases in capacity load in healthy individuals [repeated-measures ANOVA: *F*_(3,103)_ = 15.55, *p* < 10^−7^, partial η^2^ = 0.31; within-participant contrast (linear trend): *F*_(1,105)_ = 42.33, *p* < 10^−8^, partial η^2^ = 0.29], but not in the patients [repeated-measures ANOVA: *F*_(3,29)_ = 1.54, *p* = 0.226, partial η^2^ = 0.14; within-participant contrast (linear trend): *F*_(1,31)_ = 3.36, *p* = 0.076, partial η^2^ = 0.098]. As expected, the group comparison showed a significantly lower level of the measure at Load 7 in the patients with ADHD (two-sample *t* test: *t*_(136)_ = −2.17, *p* < 0.05, Cohen’s *d* = −0.44). These results support the hypothesis whereby executive dysfunction during a higher resource-demanding task in patients with ADHD may result from insufficient resource allocation of neural resources.

## Discussion

Attention is considered a central process in task-relevant information selection. Its task-selective process has been studied intensively for decades (Kanwisher and Wojciulik, 2000; Fries et al., 2001; Gazzaley et al., 2005; Falkner et al., 2010; Störmer and Alvarez, 2014; Moore and Zirnsak, 2017), while its task-general process has received less attention (Giesbrecht et al., 2003; Macaluso et al., 2003; Greenberg et al., 2010). The nonuniform allocation of limited neural resources has been suggested to be the neural basis of the broad-task process of sustained attention, but this hypothesis lacks quantitative evidence (Moray, 1967; Kahneman, 1973; Wickens, 1980; Luck et al., 1996; Wickens, 2002; Gazzaley et al., 2005; Falkner et al., 2010; Störmer and Alvarez, 2014; Watanabe and Funahashi, 2014). In the present study, we propose a measure that can provide quantitative evidence that the nonuniform allocation of neural resources reflects the broad-task process of attention. The most significant assumption is that the brain needs default amounts of neural resources that differ across brain regions to maintain the default mode (Fig. 1*a*). Based on this premise, the measure for neural resource allocation was calculated as BOLD signal changes from the resting state to task states. Finally, the nu-NRA was defined as the nonuniformity of the allocation across the whole cerebral gray matter (Fig. 1*b*). We first validated the nu-NRA using a working memory task with different difficulty levels. Task difficulty is an experimental indicator of attentional load (Sunaert et al., 2000; Culham et al., 2001; Chen et al., 2008), if the attentional load is controlled without changing types of stimuli and tasks. Participants pay more attention during difficult tasks to gain more task-relevant information to respond correctly. Gradual increases in the nu-NRA were observed, along with increases in working memory load (Fig. 2*b*). This indicates that increased attention may require a more nonuniform allocation of neural resources. The temporal dynamics of the measure showed the typical neural activity of sustained attention (Fig. 2*c*), which represents task initiation and task maintenance activity (Visscher et al., 2003; Dosenbach et al., 2006; Petersen and Dubis, 2012). We adopted an additional conventional indicator of attention level to ensure robust validation. The nu-NRA exhibited positive correlations with pupil dilation (Fig. 3), demonstrating that it accurately reflects the level of attention. We further performed cross-task and cross-dataset validations. We chose the HCP data as a validation dataset because of many subjects and many types of tasks. nu-NRA levels reflected the task difficulty well across different tasks whose attentional load was controlled without changing types of stimuli and tasks (Fig. 4). We hypothesized that broad-task neural substrates of nonuniform resource allocation might exist. We performed brain mapping and found that the broad-task neural correlates of the nu-NRA belong to frontoparietal and dorsal attention networks as well as a visual network (Fig. 5). We finally applied the measure to obtain supporting results for an existing hypothesis about the neural basis of executive dysfunction in patients with ADHD (Gualtieri and Johnson, 2006; Kratz et al., 2011; Dörrenbächer and Kray, 2019). According to this hypothesis, patients with ADHD experience an alteration in the allocation of sufficient resources to brain regions. The patients in our study exhibited low task accuracy and abnormal decreases in the nu-NRA level during the highest-load tasks (Fig. 6). This finding suggests that executive dysfunction in ADHD may be caused by alterations in resource allocation.

### Task difficulty and the nu-NRA level

Task difficulty reflecting task load has been used as an experimental inducer or indicator of attention level (Sunaert et al., 2000; Culham et al., 2001; Chen et al., 2008). In the *n*-back working memory tasks, we only induced different memory loads without changing the types of stimuli or tasks. The nu-NRA level had a positive linear relationship with task difficulty during the tasks, introducing different task loads with the same stimuli in the present study (Figs. 2*a*,*b*, 4*a–c*, 6). However, the levels were lower during the conditions with greater task difficulty in the emotion and social tasks (Fig. 4*d*,*e*). They introduced the same task load with different stimuli, not different task loads with the same stimulus for the conditions. Thus, task difficulty may be influenced by stimulus type rather than the task load. Emotional faces and interactively moving objects are more salient stimuli capturing more attention (Schupp et al., 2007; Pratt et al., 2010; Kerzel and Schönhammer, 2013). They may attract more resources for task-relevant brain regions and enhance task performance. Thus, the levels of the nu-NRA would be higher despite the lower task difficulty. This demonstrates that the nu-NRA reflects the general level of sustained attention, not just task difficulty itself.

### Retinotopy of the nu-NRA map

The nu-NRA maps for the working memory tasks reflected retinotopy in the visual cortex (Engel et al., 1997; Raz and Levin, 2014), corresponding to their stimuli (Fig. 5*a*). In Dataset 1, the stimuli were presented in the peripheral visual field, and the medial parts of the primary visual cortex showed strong positive correlations. Interestingly, the lateral parts showed negative correlations. This may be a consequence of surround suppression by lateral attention (Falkner et al., 2010; Störmer and Alvarez, 2014). By contrast, the stimuli for the working memory task in Dataset 2 were presented at the center, and the lateral parts had strong positive correlations with the nu-NRA. Furthermore, object-responsive lateral occipital regions also showed positive correlations (Grill-Spector et al., 2001; Grill-Spector and Sayres, 2008). We statistically confirmed our subjective observations by directly comparing the nu-NRA maps between datasets (two-sample *t* tests, FDR-corrected, *p* < 0.05; Extended Data Fig. 5-3). These results correspond with the retinotopy of visual-spatial attention (Tootell et al., 1998; Brefczynski and DeYoe, 1999). This further supports the notion that the nu-NRA, which quantifies the overall nonuniformity of resource allocation, temporally reflects the attention level.

### The nu-NRA as a neuromarker of general attention level

Our successful cross-task and cross-dataset validations highlighted that the nu-NRA could be used as a neuromarker of attention level in a wide range of tasks. The computation of the nu-NRA is consistent regardless of task designs or imaging protocols. It does not require any task information like onsets of cues and stimuli for the computation. We could rather predict the onsets from the temporal dynamics of the nu-NRA (Fig. 2*c*). The generality and utility of the measure allow researchers to apply this method to their existing fMRI dataset easily. The neuromarker may help quantify the universal level of attention in multiple-task paradigms, especially in multisensory research. The mechanism of attentional resource allocation is still a part of the debate about whether the resources are shared across sensory modalities (Soto-Faraco and Spence, 2002; Chan and Newell, 2008; Finoia et al., 2015; Wahn and König, 2015a,b, 2017). Previous research has investigated whether the task performance decreases during a dual-task condition compared with a single-task condition under an assumption that the overall resource demands are consistent across conditions. Thus, the neuromarker would provide a quantitative validation for the assumption in multisensory research. The neuromarker may also help to investigate the temporal effects of nonuniform resource allocation on other cognitive processes. Arousal and attention processes are highly interactive with each other (Posner, 1994; Harth, 1995; Coull, 1998; Portas et al., 1998; Foucher et al., 2004). Thus, their effects are difficult to separate. Suppose the nu-NRA can be used as a quantitative proxy of attention level with an fMRI arousal index (Chang et al., 2016). In that case, we could temporally distinguish their independent and interactive effects on various behavioral and neural phenomena. This will lead to the specification of the neural basis of attention and arousal. Furthermore, the nu-NRA could be an additional parameter to the computational models of other neural processes, such as perceptual learning or hierarchical predictive coding (Lu et al., 2011; Bastos et al., 2012; Dosher and Lu, 2017). This would aid in understanding the role of the resource allocation process in human cognition.

### Study limitations

The present study has some limitations, despite the robust validation on the neural basis of broad-task sustained attention. First, the computation of the nu-NRA could be affected by the length of resting-state fMRI scans. It is well known that there is BOLD signal variation across the resting state. Thus, the short duration may poorly estimate the default resource utilization by averaging. We did not validate the optimal duration for the estimation, but we used several proper durations (3 min for Dataset 1, 15 min for Dataset 2, and 5 min for Dataset 3) according to previous studies (Whitlow et al., 2011; Birn et al., 2013). However, the effect of the length of the resting state remains to be further explored. In addition, a comparison between resting conditions, eyes open, closed, and fixated is necessary because of their differences in resting-state fMRI features (Patriat et al., 2013; Agcaoglu et al., 2019). Second, the nu-NRA level is sensitive to protocol types that direct comparison of nu-NRA levels between different datasets. However, the normalized nu-NRA level could be compared between subjects using the same experimental design. Third, the level of the nu-NRA did not solely reflect the task difficulty in several tasks. In Dataset 2, the difference the level of the nu-NRA between different conditions from the relational task, which introduced matching and relation trials with different attentional load without changing types of stimuli, showed a marginal significance (Fig. 4*b*). This result may be caused by the longer processing times (>500 ms) for relation trials than matching trials. Relation trials asked participants to do multistep processing. They first found a relation between each pair and then compared the relations between different pairs. Thus, the task difficulty may depend on how long the attention is maintained for a certain level rather than how much greater the attention level is. However, the computation of the nu-NRA level would reflect how much greater the attention level is rather than how long the attention is maintained. In Dataset 3, the nu-NRA level reflected the trend of the task accuracy but not the exact differences between the task conditions (Fig. 6). The level showed minor differences between lower-capacity loads, but task accuracy showed significant differences in both groups. In addition, the between-group analysis showed lower accuracy with no difference in the level of the measure during Load 5. This result indicated that other factors, such as problems at a memory-encoding stage, might cause task performance differences in the resource allocation process. Alterations in the memory encoding may not be reflected in the measure. Further studies are necessary to develop multidimensional measures for measuring sustained attention levels to validate these mismatches.

## Synthesis

Reviewing Editor: Alexander Soutschek, Ludwig-Maximilians-Universitat Munchen Decisions are customarily a result of the Reviewing Editor and the peer reviewers coming together and discussing their recommendations until a consensus is reached. When revisions are invited, a fact-based synthesis statement explaining their decision and outlining what is needed to prepare a revision will be listed below. The following reviewer(s) agreed to reveal their identity: Ayumu Yamashita, Agnieszka Zuberer.

The reviewers appreciate the authors’ explanations and a lot of additional analyses to answer their questions. The authors have addressed almost all of the concerns. However, the reviewers are still concerned regarding their argument about generality of the nu-NRA. “Task-general attention” seems too strong to say from their results. Their main results to support generality were Fig4, Fig5b and c. However, the interpretation of the nu-NRA level differences between conditions in emotion and social tasks were based on their speculation. Furthermore, high cross-task spatial similarity in Fig5b were mainly driven by visual region (Fig5c showed this). The reviewers understand that the bilateral parietal and right middle frontal regions were overlap across tasks, but the visual regions were obviously dominant. From those reasons, the reviewers are not convinced that the nu-NRA is a neuromarker of the “task-general attention”. Therefore, the authors should change the strong word “task-general attention” to weaker word and tone down the generality of nu-NRA in the Discussion (e.g. p16 line 377- p17 line 387 and p19 lines 454-457).

Minor comments

1. From above reason, the authors may want to exclude Fig5b, which could mislead readers, and show nu-NRA maps for all tasks in the main text or supplementary information.

2. p17 lines 409-410 “We performed brain mapping and found that the task-general neural correlates of the nu-NRA belong to fronto-parietal and dorsal attention networks (Figure 5).” This is misleading. The overlaps were mainly in visual region.

3. Please report effect size estimates (e.g. Cohen’s d for t-test, partial eta square for ANOVAs) for all statistical tests.
