# Supplementary material for: Nonuniformity of Whole-Cerebral Neural Resource Allocation, a Neuromarker of the Broad-Task Attention
Source: eNeuro. 2022 Mar 14;9(2):ENEURO.0358-21.2022. doi: 10.1523/ENEURO.0358-21.2022 (PMC8925723; doi:10.1523/ENEURO.0358-21.2022)
Supplement: Extended Data 1 — Supplementary Code_Data_Accessibility. Download Extended Data 1, DOCX file. [file enu-eN-NWR-0358-21-s08.docx]

**Data and Code Accessibility Statement**

1. **Visuospatial n-back working memory task (Dataset 1).**

- You can download this dataset from Jinyong Chung’s data repository, Mendeley Data (“WorkingMemoryfMRI_KAIST/rawData.zip”).
- The dataset was approved by the Institutional Review Board of KAIST. Twenty-four participants [four women; age range: 19-34 years (mean age = 25 years); all right-handed] were recruited from the Korea Advanced Institute of Science and Technology (KAIST). No participant had any history of neurological/psychiatric illness, and all had 20/20 vision. One participant (number 9) was excluded from all analyses, as the upper part of the brain was out of the fMRI field-of-view. Task performance and physiological data were missing in six participants (number 1~5 and 13), who were thus excluded from the corresponding analyses.
- We collected 3 minutes of resting-state fMRI (eyes open) and structural T1 MRI data before the fMRI task.
- Participants performed passive viewing, 1-, 2-, and 3-back tasks while attending to the left or right side of the stimulus during the fMRI acquisition. The task order is saved in “TaskOrder.m” file. Each row indicates each participant’s task order (“1” = passive viewing task, “2” = 1-back task, “3” = 2-back task, and “4” = 3-back task).
- Number 1~5 subjects did a little different version of the experiment. After a 60-s fixation block, four tasks were performed by following the task order. Each task cues were presented for 3 s, followed by a 1-s delay. Cues consisted of a number representing the n-back type and an eye icon. The left n-back task began after 3 s of “left” spatial cue presentation and a 1-s delay. After the left n-back task, the right n-back task began following the presentation of a “right” spatial cue. Forty trials were included for each spatial n-back task. Each trial included 2 s of stimulus presentation followed by a 0.5-s delay. At the end of each task, an additional 30-s fixation block was performed.
- The others did the same version of the experiment, which was depicted in the supplementary materials section.
- Example data and code for the nu-NRA computation are included in “nuNRA_DATA&Code.zip”.

1. **Human Connectome Project data (Dataset 2).**

- We selected and downloaded 98 participants (68 women; age range: 22-35 years) of the Human Connectome Project dataset (<https://db.humanconnectome.org/>), referring to the list of HCP participants provided by Tavor et al. (Tavor et al. 2016).
- See a reference: Tavor, I., Jones, O.P., Mars, R.B., Smith, S.M., Behrens, T.E., Jbabdi, S., 2016. Task-free MRI predicts individual differences in brain activity during task performance. Science 352, 216-220. https://doi.org/10.1126/science.aad8127.

1. **The Consortium for Neuropsychiatric Phenomics (CNP) database (Dataset 3).**

- The CNP database is a shared neuroimaging dataset that focuses on exploring memory and cognitive control in patients with neuropsychiatric disorders [healthy individuals (n=130), schizophrenia (n=50), bipolar disorder (n=49), and attention-deficit/hyperactivity disorder (ADHD) (n=43)]. The database can be downloaded from OpenfMRI project (https://legacy.openfmri.org/dataset/ds000030/). We selected 106 healthy individuals [50 women; age range: 21-50 years (mean age = 32 years); all right-handed] and 34 patients with ADHD [19 women; age range: 21-50 years (mean age = 35 years); all right-handed] with no aliasing artifacts on T1 images.
- See a reference: Poldrack, R.A., Congdon, E., Triplett, W., Gorgolewski, K.J., Karlsgodt, K.H., Mumford, J.A., Sabb, F.W., Freimer, N.B., London, E.D., Cannon T.D., Bilder R.M., 2016. A phenome-wide examination of neural and cognitive function. Sci. Data 3, 160110. https://doi.org/10.1038/sdata.2016.110.
