# Supplementary figures and images for: Nonuniformity of Whole-Cerebral Neural Resource Allocation, a Neuromarker of the Broad-Task Attention
Source: eNeuro. 2022 Mar 14;9(2):ENEURO.0358-21.2022. doi: 10.1523/ENEURO.0358-21.2022 (PMC8925723; doi:10.1523/ENEURO.0358-21.2022)

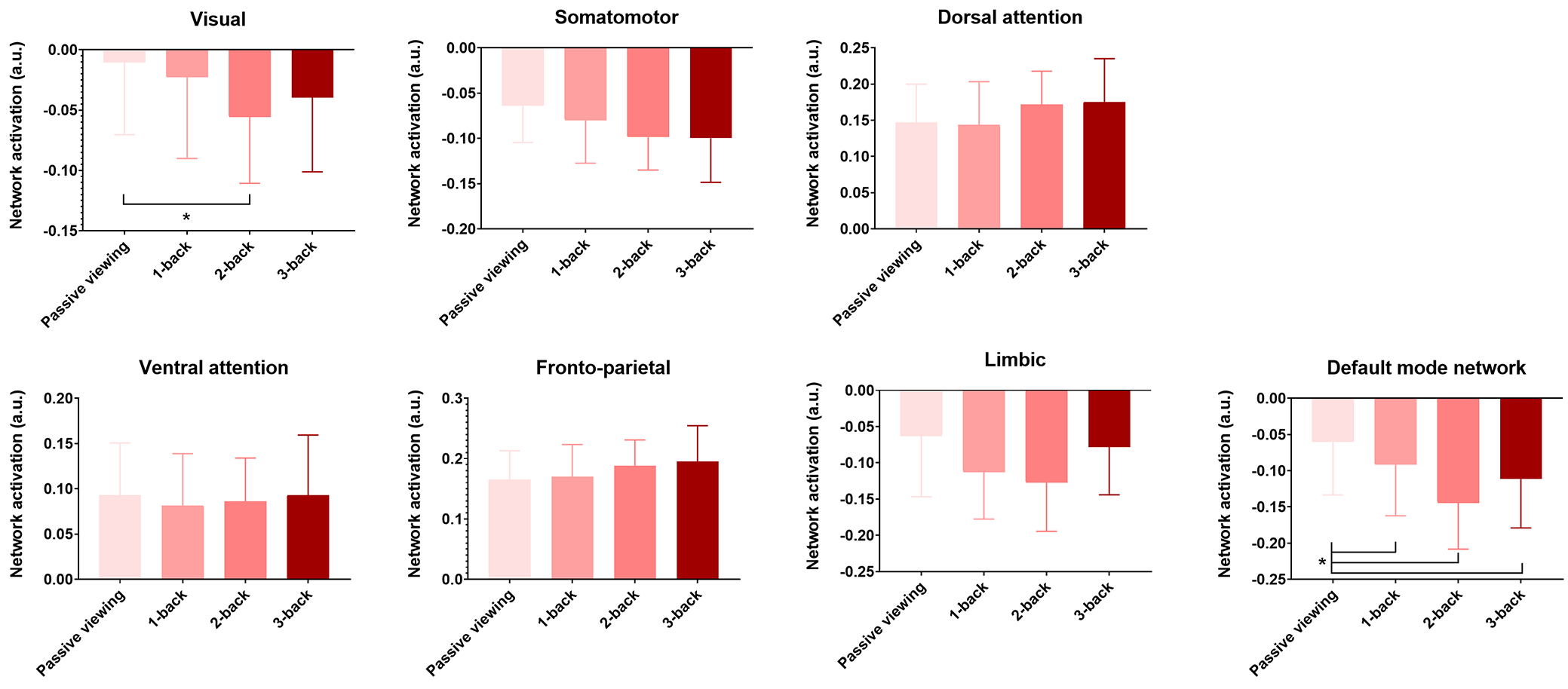

Supplement: Figure 2-1 — Network activations during the visuospatial n-back working memory task in Dataset 1. *p < 0.05, **p < 0.01, ***p < 0.001. Download Figure 2-1, TIF file. [file enu-eN-NWR-0358-21-s02.tif]

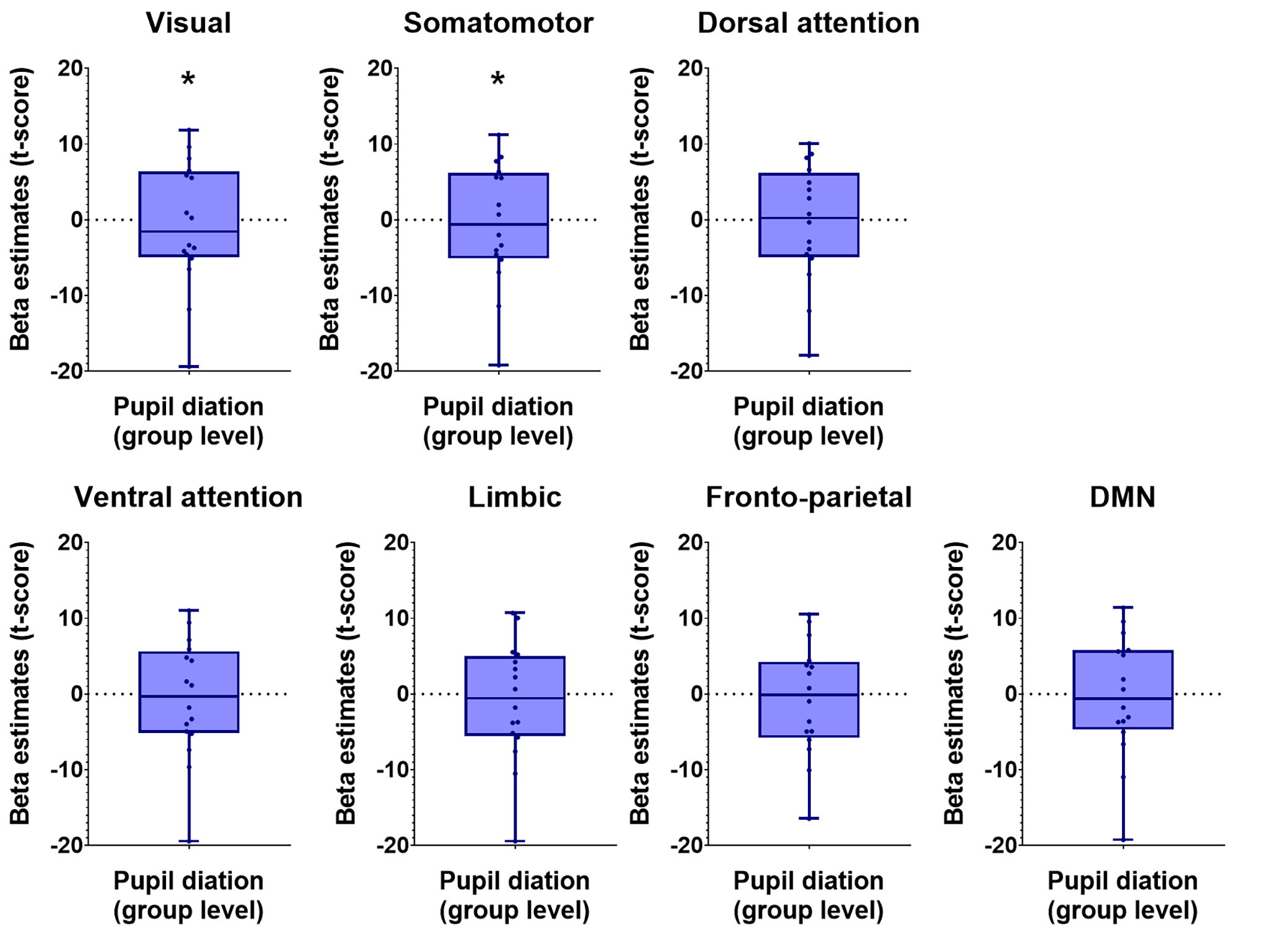

Supplement: Figure 3-1 — Relationship between network activations and pupil dilation. *p < 0.05, **p < 0.01, ***p < 0.001. Download Figure 3-1, TIF file. [file enu-eN-NWR-0358-21-s03.tif]

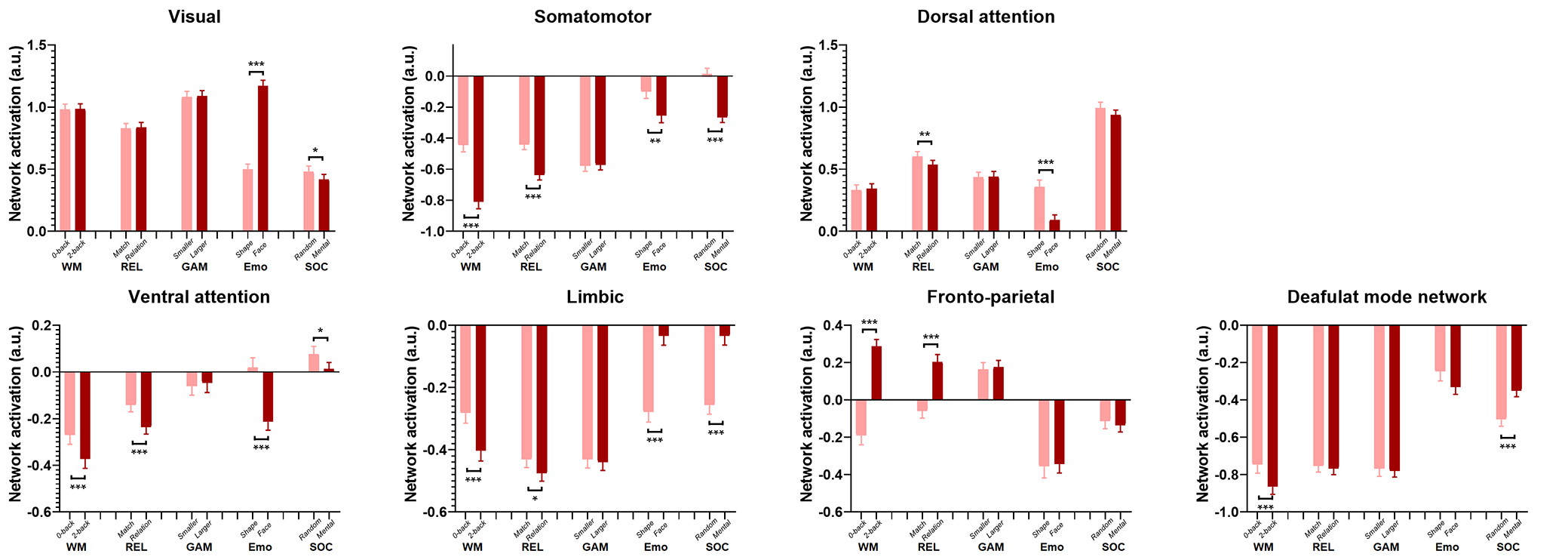

Supplement: Figure 4-1 — Network activations across task conditions in Dataset 2. *p < 0.05, **p < 0.01, ***p < 0.001. Download Figure 4-1, TIF file. [file enu-eN-NWR-0358-21-s04.tif]

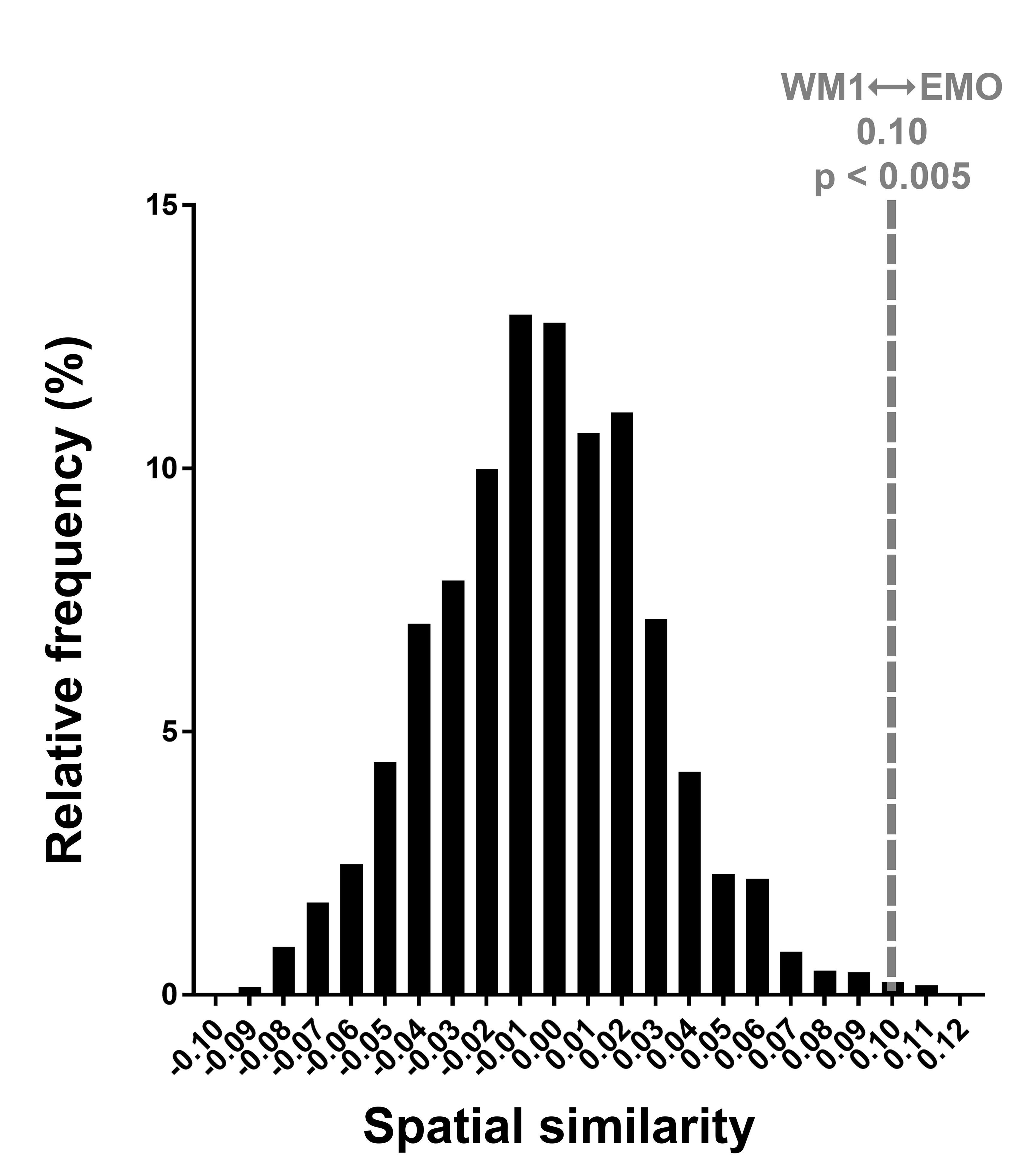

Supplement: Figure 5-1 — Randomization distribution for spatial similarities among the group nu-NRA maps. The mean value of permuted spatial similarities is near zero, and the maximum permuted similarity is near 0.11. The p value for the spatial similarity (r = 0.10) between the working memory task from Dataset 1 (WM1) and the emotion task from Dataset 2 (EMO) is <0.005. Download Figure 5-1, TIF file. [file enu-eN-NWR-0358-21-s05.tif]

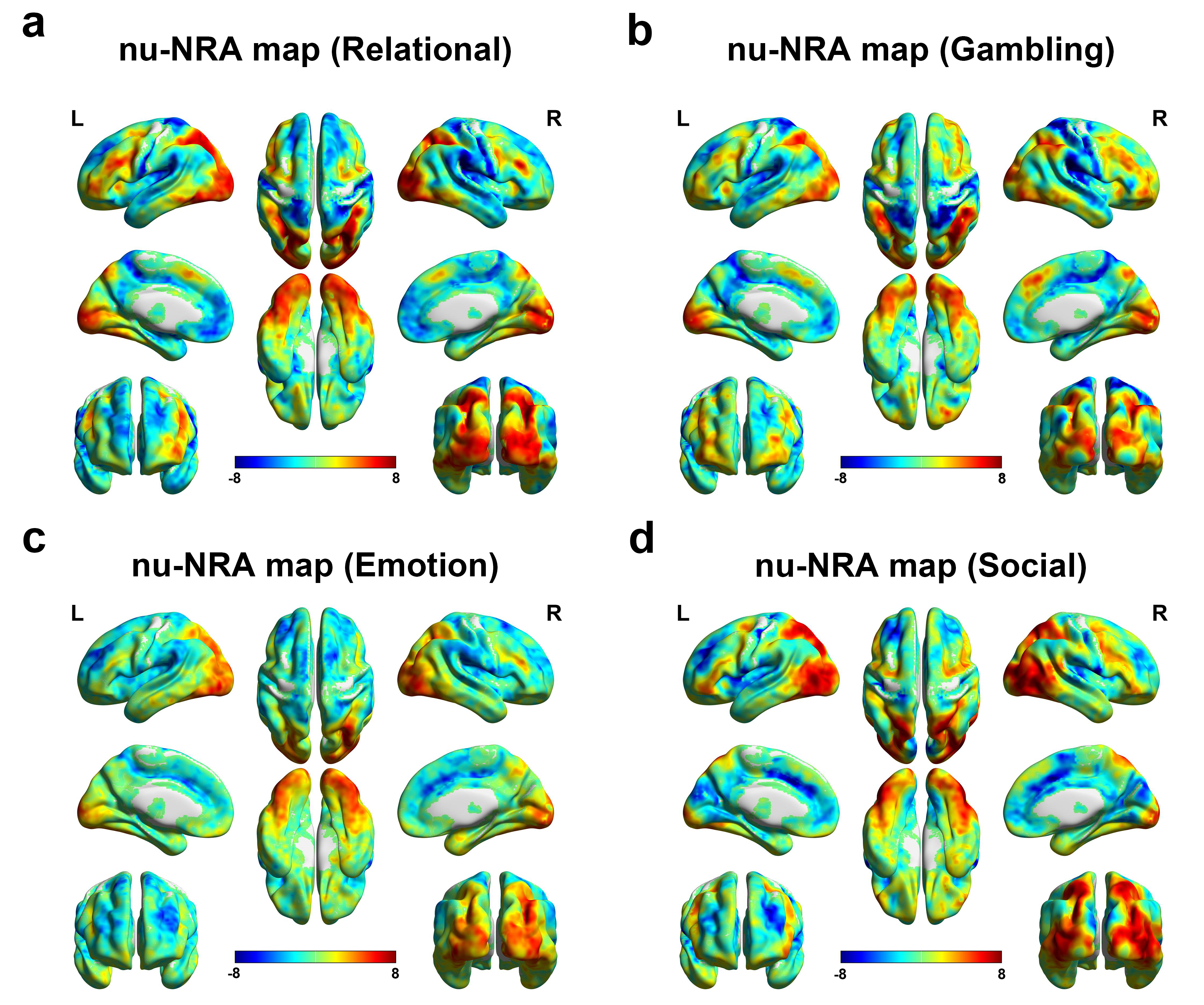

Supplement: Figure 5-2 — nu-NRA maps for four tasks in Dataset 2. a, Relational task. b, Gambling task. c, Emotion task. d, Social task. Download Figure 5-2, TIF file. [file enu-eN-NWR-0358-21-s06.tif]

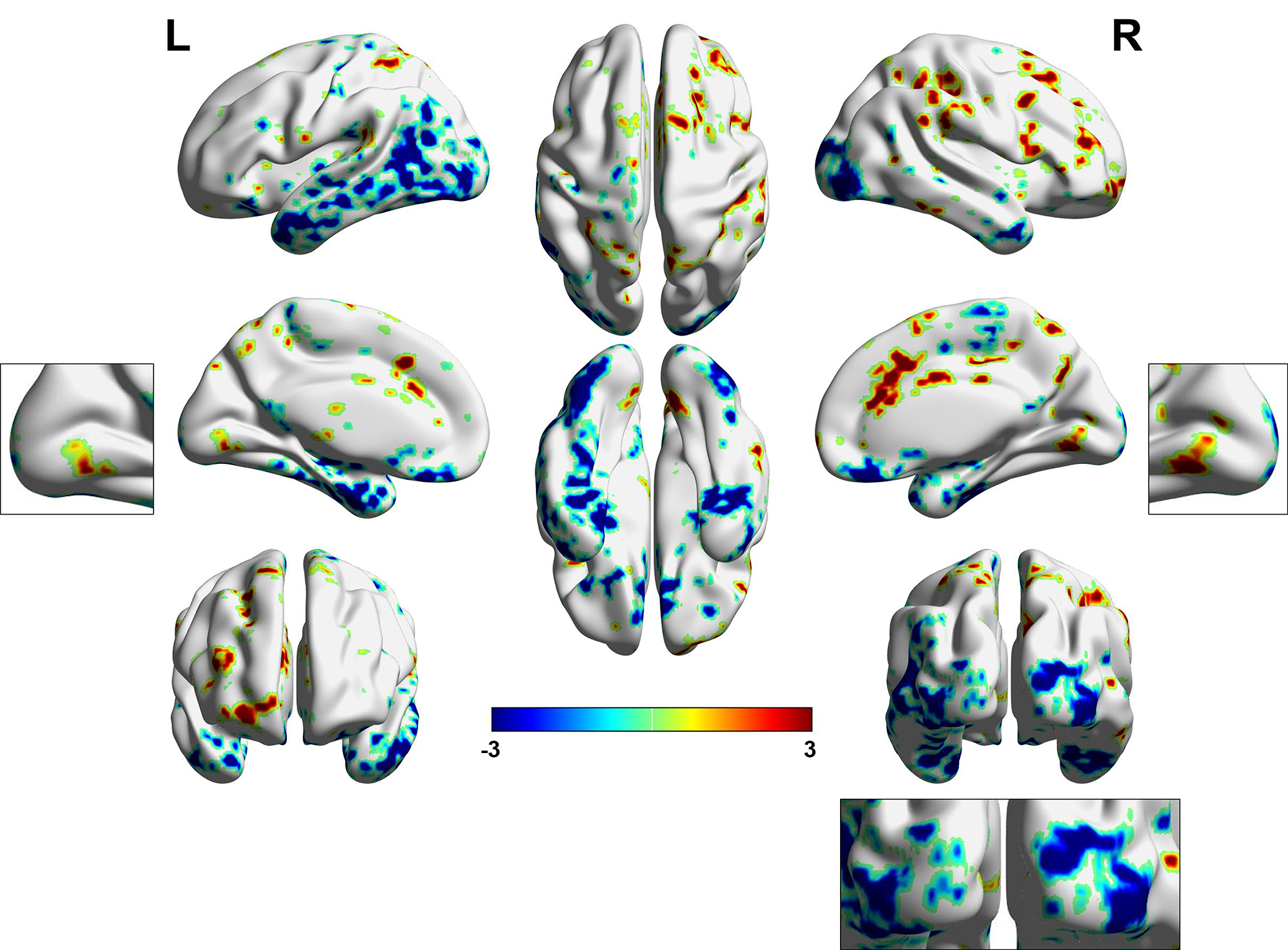

Supplement: Figure 5-3 — Comparison of the nu-NRA maps during the working memory tasks between Dataset 1 and 2. Positive (red) values indicate higher correlations (two-sample t tests, FDR-corrected, p < 0.05) in Dataset 1 than in Dataset 2. Dataset 1, presenting peripheral stimuli, shows higher correlations in medial parts of the primary visual cortex. By contrast, Dataset 2, presenting central object stimuli, shows higher correlations (blue) in the lateral parts of the primary visual cortex and object-responsive lateral occipital regions. Download Figure 5-3, TIF file. [file enu-eN-NWR-0358-21-s07.tif]
